# Flexible model of network embedding

**DOI:** 10.1038/s41598-019-48217-x

**Published:** 2019-08-12

**Authors:** Juan Fernández-Gracia, Jukka-Pekka Onnela

**Affiliations:** 1 Department of Epidemiology, Harvard T.H. Chan School of Public Health, Harvard University, Boston, MA 02115 USA; 2 Instituto de Física Interdisciplinar y Sistemas Complejos IFISC (CSIC - UIB), Palma de Mallorca, E-07122 Spain; 3 Department of Biostatistics, Harvard T.H. Chan School of Public Health, Harvard University, Boston, MA 02115 USA

**Keywords:** Complex networks, Applied mathematics

## Abstract

There has lately been increased interest in describing complex systems not merely as single networks but rather as collections of networks that are coupled to one another. We introduce an analytically tractable model that enables one to connect two layers in a multilayer network by controlling the locality of coupling. In particular we introduce a tractable model for embedding one network (A) into another (B), focusing on the case where network A has many more nodes than network B. In our model, nodes in network A are assigned, or embedded, to the nodes in network B using an assignment rule where the extent of node localization is controlled by a single parameter. We start by mapping an unassigned “source” node in network A to a randomly chosen “target” node in network B. We then assign the neighbors of the source node to the neighborhood of the target node using a random walk starting at the target node and with a per-step stopping probability *q*. By varying the parameter *q*, we are able to produce a range of embeddings from local (*q* = 1) to global (*q* → 0). The simplicity of the model allows us to calculate key quantities, making it a useful starting point for more realistic models.

## Introduction

The last decade has witnessed a transition from the study of simple graphs to more complex structures^1^, such as bipartite networks, temporal networks^2^, networks of networks^3^, and multiplex and multi-layer networks^4^. All these entities have been shown to be of interest for the modeling of different systems at different levels of complexity^5–7^. In the context of multi-layer networks, although there are many generative models for the individual layers that compose them (see for example^1,8^), the investigation of such models for inter-layer connectivity is scarce and researchers have explored mostly correlations of different centrality measures to establish inter-layer connections, such as by connecting high-degree nodes in one layer to low-degree nodes in the other layer^9–11^. There are naturally many other ways how nodes in different layers may be coupled, and we introduce a generative and analytically tractable model of inter-layer connectivity that allows for controlling the extent to which locality of connections in one layer is preserved when the network is embedded to the nodes of another layer. We start with a model that consists of pairwise interconnections between layers. We use the term “embedding” to refer to an inter-layer connectivity pattern where all nodes of one of the two layers, layer or network A, have at least one link to a node in the other layer, layer or network B), *i.e*., the coupling is an exhaustive mapping of the nodes of network A to the nodes of network B. Throughout the manuscript, we will use the language of embedding rather than that of multilayer networks. This type of embedding appears naturally in various settings. For example, network A could correspond to a social network of individuals and their pairwise social relationships and network B could represent a network of spatial locations (counties, cities, etc.) and their pairwise connectivity (spatial adjacency, transportation network, etc.).

Note that *embedding* here refers to a different concept than metric embedding of a network into a metric space^12^ as we do not aim to have a metric space representation of a network which can substitute the topology of the network and be used, for example, for routing purposes using only local knowledge about the structure of the network^13^.

Throughout this paper, we will think of network A as representing a social network and network B a network of geographical locations. Of key interest is the extent of the locality of the embedding. After a “source” node in A has been mapped to a “target” node in B, locality of the embedding refers to the extent to which the neighbors of the source node in A are mapped to the neighborhood of the target node in B. This characteristic has been studied for social networks embedded in space, finding that the probability of having a social contact at a certain distance is usually well described by a power law with exponent varying between −0.7 and −2^14–17^. For this specific setting there are existing models that couple mobility and social connections in order to reproduce specific features of the social networks embedded in geography, such as the similarity of visitation patterns for neighbors in the social network^18^, the degree distribution of social networks and the spatial spread of populations^19^, or the coupled evolution of mobility and social interactions^14^.

This varying degree of locality has important consequences for processes that take place on a network that is embedded in space. For example, for epidemic processes one can transition from a situation of local embedding, in which spreading fronts move at a well defined velocity over space, to a situation where there are no spreading fronts at all and the spatial description of the epidemics cannot be tackled by a continuous space approach^20^. Being able to model this characteristic, the extent of locality of the network embedding, can help one understand emergent properties of dynamical models involving opinion dynamics, epidemic spreading, or diffusion of innovations or cultural traits.

## The Model

We consider two undirected and unweighted networks *G*_*A*_ and *G*_*B*_ with *N*_*A*_ and *N*_*B*_ nodes, respectively, and we assume that *N*_*A*_ > *N*_*B*_. Their degree distributions are *P*_*A*_(*k*) and *P*_*B*_(*k*) and their adjacency matrices are ***A*** and ***B***. The assignment rule embeds network A in network B, i.e., each node in *G*_*A*_ will be assigned to a node in *G*_*B*_. Completion of the node assignment process gives rise to a third network called the *embedded network G*_Γ_, which is an undirected weighted network with adjacency matrix **Γ** consisting of the same same set of nodes as *G*_*B*_. Some of the nodes in *G*_*A*_ will be assigned to node *i* in *G*_*B*_ and some to node *j* in *G*_*B*_; the element Γ_*ij*_ of the adjacency matrix of the embedded network corresponds to the number of edges in *G*_*A*_ between the group of nodes assigned to node *i* in *G*_*B*_ and the group of nodes assigned to node *j* in *G*_*B*_. We use *f*_*i*_ to denote the *attractiveness* of node *i* in *G*_*B*_, which is a measure of its ability to attract nodes from network A, and we impose the condition that the attractiveness of all nodes add up to 1 (${\sum }_{i=1}^{{N}_{B}}\,{f}_{i}=1$) to allow for its interpretation as probability. The values *f*_*i*_ can be assigned based on any rule, which might be coupled to network structure; for example, they could be made proportional to the degrees of nodes in network B or they could depend on an exogenous factor. Returning to the example of a social network A that is embedded in a spatial network B, the attractiveness of nodes in network B could be thought of as the target proportions of agents to embed at each node. Depending on the application, this could reflect the capacities of nodes in network B, which might be proportional to, say, populations of cities. We would like the network embedding process to be carried out such that by varying some coupling parameter, the resulting embedded network *G*_Γ_ has a varying degree of locality, which leads us to the following simple assignment rule which is repeated until all nodes in *G*_*A*_ have been assigned to nodes in *G*_*B*_.

1. Choose at random an unassigned node *α* in *G*_*A*_ (source node) and assign it to node *i* in *G*_*B*_ (target location) with probability *f*_*i*_.
2. For each unassigned neighbor of the source node *α* in *G*_*A*_, start a weighted random walk from the target node *i* in *G*_*B*_ with a per-step stopping probability *q* (probability that the random walk stops at each step); assign the node in *G*_*A*_ to the node in *G*_*B*_ where the random walk happens to stop. The random walk on *G*_*B*_ is weighted at each node by the attractiveness of the neighboring (adjacent) nodes.

**Figure 1:**
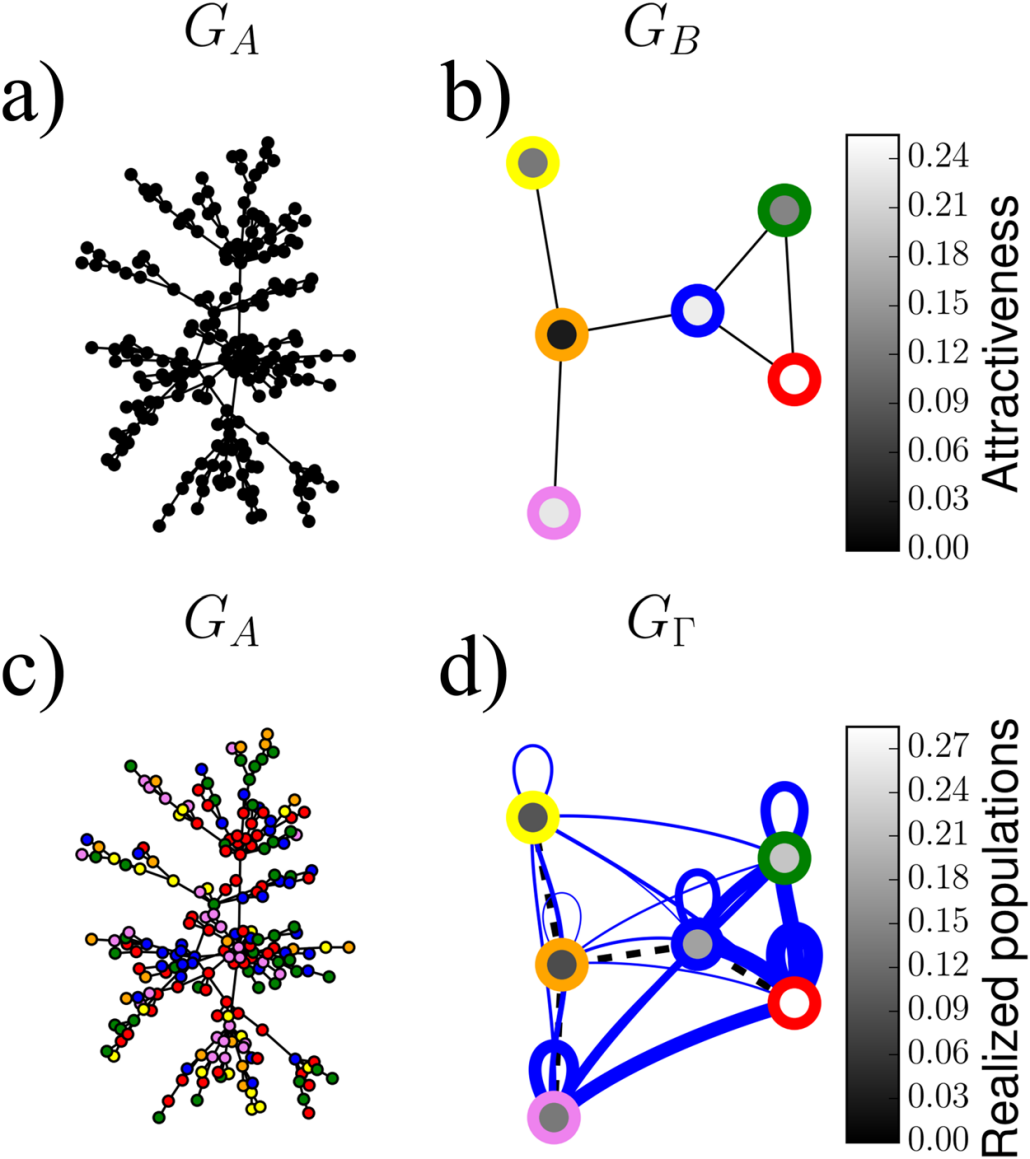
Example of the network embedding model. (**a**) Visualization of network A, here a BA network^23^ of size *N*_*A*_ = 100 and parameter *m* = 1. (**b)** Network B is very simple for illustrative purposes. The outside coloring of each node represents its label and the inside color represents its attractiveness. (**c)** The nodes in A have been assigned to location nodes in B and the color of each network A node matches the outside coloring of a network B node. (**d)** Embedded network ${G}_{\Gamma }$. The outside coloring of each node is the same as in *G*_*B*_ whereas the inside color represents the percentage of network A nodes that have been assigned to the given node in network B.

If the stopping probability *q* = 1, the walk always (deterministically) comes to a halt at the location node *i* in *G*_*B*_ where it started, and therefore all unassigned neighbors of a given source node are assigned to the same location as the source node itself. This results in an embedded network *G*_Γ_ that represents the strongest possible coupling between networks A and B. The value of *q* = 0 for the stopping probability is not sensible in the context of the proposed model because after assigning a source node, no neighboring nodes would ever be assigned (stopping probability is 0). However, we can consider the limit of *q* → 0 in which case we simply assign unassigned neighbors of a source node *α* in *G*_*A*_ to a location node *i* in *G*_*B*_ with probability that is proportional to the stationary probability of a biased random walker to be at any of the nodes. The probability of being assigned to node *i* in B will thus be proportional to the corresponding entry of the leading eigenvector ***v***^0^ with eigenvalue 1 of the transition matrix ***C*** (${C}_{ij}={f}_{i}{B}_{ij}/{\sum }_{k}\,{f}_{k}{B}_{kj}$), which describes the weighted random walk on network B^21^. In this case the embedded network *G*_Γ_ represents the weakest possible coupling between networks A and B. For intermediate values of 0 < *q* < 1 the extent of embedding interpolates between these two extreme cases^22^. For a schematic of the model, see Fig. 1.

## Analytical Description

The assignment process stops once every node in *G*_*A*_ has been assigned to a location node in *G*_*B*_. At this point there are several quantities of interest that describe the output of the model, and these include the size of the realized population Φ_*i*_ at each node *i* in *G*_*B*_, which corresponds to the number of nodes in *G*_*A*_ that have been assigned to location node *i*, and the adjacency matrix **Γ** of the embedded network *G*_Γ_. We restrict the analytics to the case where network A has many more nodes than network B, and therefore for network A we make use of the degree distribution *P*_*A*_(*k*) only whereas for network B we use the actual adjacency matrix ***B***. Note that prior to calculating the sizes of the realized populations Φ_*i*_ at location nodes, or the adjacency matrix of the embedded network **Γ**, we can describe analytically the number of nodes in network *G*_*A*_ that have been assigned to a location node in *G*_*B*_ at different times of the process and how many neighbors they have in *G*_*A*_ with either assigned or unassigned locations in network B. This process can be described independently of any knowledge of the topology of network B or the random walk stopping probability *q*, and since these details are needed for calculating other features of the model, we will start the analytics from there.

### Process of location assignment

**Figure 2:**
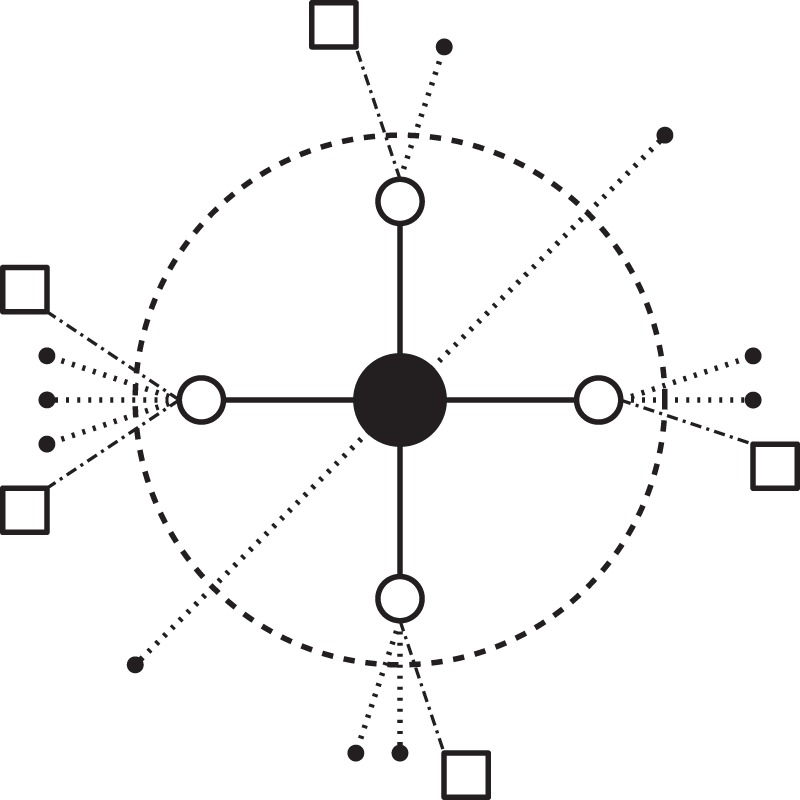
Schematic of location assignment. Shown is a piece of network A that is centered around a node that is chosen at random for location assignment during this time step (shown as the black filled node at the center). The empty circles represent unassigned neighbors of the central node, and the nodes inside the big dashed circle are the ones to be assigned during this time step. The empty squares represent unassigned second neighbors of the central node. Finally, the small filled circles represent assigned nodes. Location assignments in the random walk process will be correlated only for the nodes that are connected with solid edges.

As part of the assignment of nodes at each time step we choose an unassigned source node *α* in *G*_*A*_ at random and assign the node itself, as well as its unassigned nearest neighbors in *G*_*A*_, to target location nodes in *G*_*B*_. We are interested in two specific quantities related to unassigned nodes in *G*_*A*_ at time *t*, namely, the distribution ${P}_{A}^{\oplus }(k,t)$ of the number of their *unassigned* neighbors *k* (empty circles in Fig. 2) and the distribution ${P}_{A}^{\dagger }(k,t)$ of the number of their *assigned* neighbors *k* (small black circles in Fig. 2). We reduce the description to the computation of the first two moments of ${P}_{A}^{\oplus }(k,t)$, ${\langle k\rangle }_{A}^{\oplus }$ and ${\langle {k}^{2}\rangle }_{A}^{\oplus }$, and the first moment of ${P}_{A}^{\dagger }(k,t)$, ${\langle k\rangle }_{A}^{\dagger }$. Their approximate evolution equations are

1 $$\frac{d}{dt}{\langle k\rangle }_{A}^{\oplus }(t)=\frac{1}{\eta (t)-1-{\langle k\rangle }_{A}^{\oplus }(t)}[{\langle k\rangle }_{A}^{\oplus }{(t)}^{2}-2{\langle {k}^{2}\rangle }_{A}^{\oplus }(t)],$$

2 $$\frac{d}{dt}{\langle {k}^{2}\rangle }_{A}^{\oplus }(t)=\frac{1}{\eta (t)-1-{\langle k\rangle }_{A}^{\oplus }(t)}\times \{{\langle {k}^{2}\rangle }_{A}^{\oplus }(t)[1-2{\langle k\rangle }_{A}^{\oplus }(t)-2\frac{{\langle {k}^{2}\rangle }_{A}^{\oplus }(t)}{{\langle k\rangle }_{A}^{\oplus }(t)}]+2{\langle k\rangle }_{A}^{\oplus }{(t)}^{3}\},$$

3 $$\frac{d}{dt}{\langle k\rangle }_{A}^{\dagger }(t)=\frac{{\langle {k}^{2}\rangle }_{A}^{\oplus }(t)}{\eta (t)-1-{\langle k\rangle }_{A}^{\oplus }(t)},$$

with initial conditions $\eta \mathrm{(0)}={N}_{A}$, ${\langle k\rangle }_{A}^{\oplus }\mathrm{(0)}={\langle k\rangle }_{A}$, ${\langle {k}^{2}\rangle }_{A}^{\oplus }\mathrm{(0)}={\langle {k}^{2}\rangle }_{A}$, and ${\langle k\rangle }_{A}^{\dagger }\mathrm{(0)}=0$, where ${\langle k\rangle }_{A}$ and ${\langle {k}^{2}\rangle }_{A}$ are the first and second moments of the degree distribution of network A and $\eta (t)$ is the total number of unassigned nodes. The derivation of these equations only assumes that the network is locally tree-like and with no degree-degree correlations, and therefore we would expect it to be reasonably accurate for uncorrelated networks with low levels of clustering. For a complete derivation of the equations see the SI.

After some t^*^ time steps all nodes in network A will have been assigned, which implies that ${N}_{A}={\int }_{0}^{{t}^{\ast }}\,[1+{\langle k\rangle }_{A}^{\oplus }(t)]dt$.

We can then integrate Eqs 1–4 together with their initial conditions numerically; here we used the RK4 algorithm but other choices are also possible. The agreement is very good between direct simulations from model and numerical integration of the equations for different types of networks of different sizes (see Appendix Figs S2 and S3).

### Realized populations

We use Φ*i*(*t*) to denote the number of nodes in A that have been assigned to target location node *i* in B at time *t*. In the case $q\ne 0$, the average realized population sizes are given by (see Appendix for derivation):

4 $$\langle {{\rm{\Phi }}}_{i}\rangle ={f}_{i}\delta (q)+q\alpha \sum _{j}\,{f}_{j}\mathop{\sum }\limits_{r\mathrm{=1}}^{\infty }\,{(1-q)}^{r}{[{{\boldsymbol{C}}}^{r}]}_{ij},$$

with $\delta (q)={\int }_{0}^{{t}^{\ast }}\,[1+q{\langle k\rangle }_{A}^{\oplus }(t)]dt$, $\alpha ={\int }_{0}^{{t}^{\ast }}\,{\langle k\rangle }_{A}^{\oplus }(t)dt$ and ${C}_{ij}={f}_{i}{B}_{ij}/{\sum }_{l}\,{f}_{l}{B}_{lj}$. In the case *q* = 1, and combining the equation for the realized populations (Eq. 4), the definition of $\delta (q)$ and the implicit equation for t*, we see that $\langle {{\rm{\Phi }}}_{i}\rangle ({t}^{\ast })={N}_{A}{f}_{i}$. For intermediate values of *q*, the random walk based node assignment process distorts the realized population sizes described by Eq. 4. For the case *q* = 0, the average realized populations will be

5 $$\langle {{\rm{\Phi }}}_{i}\rangle ={f}_{i}({N}_{A}-\alpha )+\alpha {v}_{i}^{0}.$$

**Figure 3:**
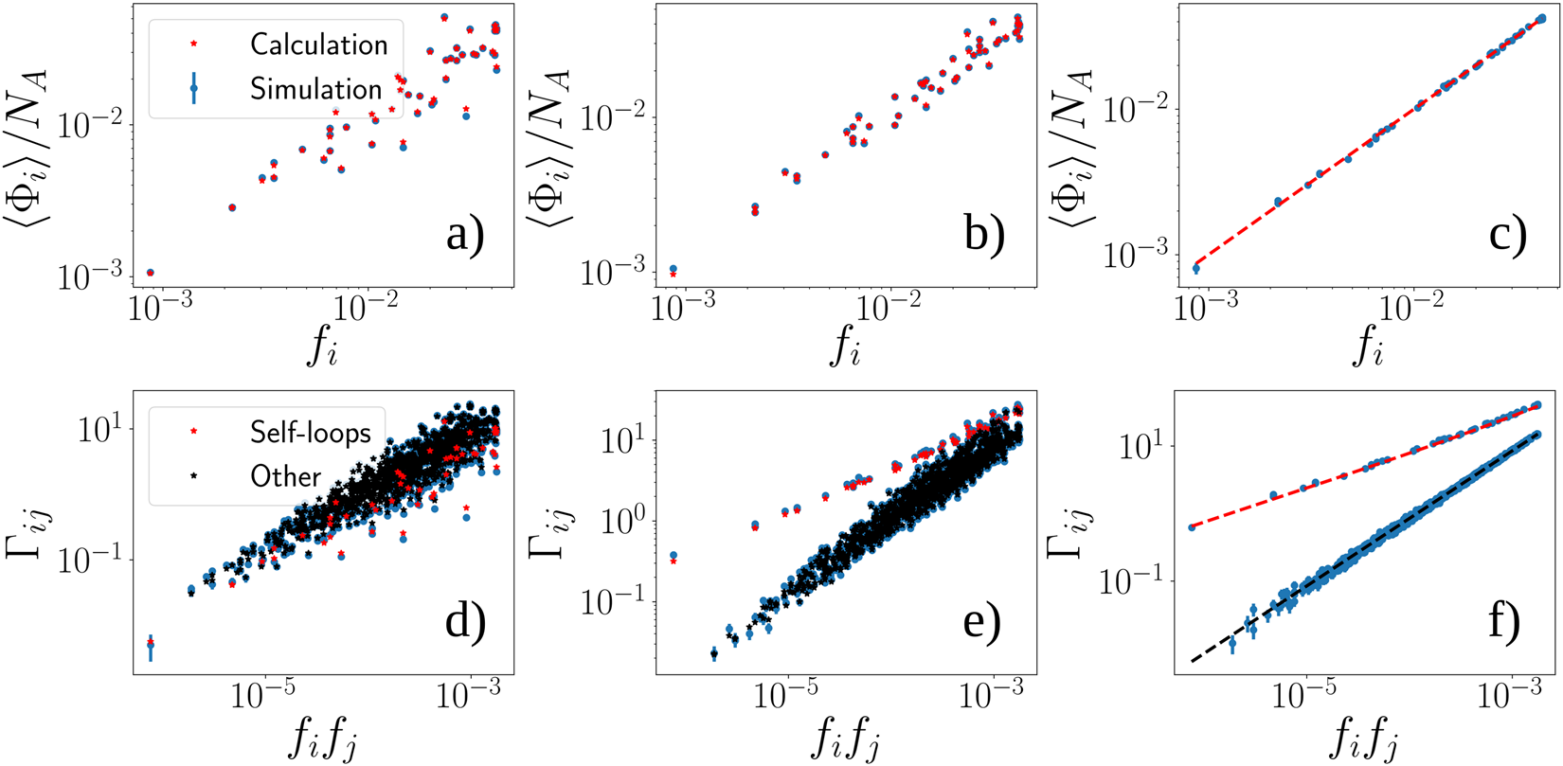
Simulation results and analytic results for the outcomes of the model. *q* = 0 (**a,d**), *q* = 0.5 (**b,e**) and *q* = 1 (**c,f**). Realized populations in the top row and weights in the bottom row. Here *G*_*A*_ corresponds to an Erdös-Reny (ER) random graph of size *N*_*A*_ = 10^3^ with edge probability of *p* = 10^−2^. We generate 1000 network realizations from the ER model and assign them to the target nodes of network B, which here corresponds to a 50-node network that are randomly scattered on a 2-dimensional square and are linked by their spatial adjacency based on a Voronoi tessellation. Results shown are an average over the 1000 independent realizations of the networks. The attractiveness of nodes *f* in *G*_*B*_ are sampled from a uniform (1, 100) distribution and then rescaled to satisfy the constraint ${\sum }_{i=1}^{{N}_{B}}\,{f}_{i}=1$. The simulated values are shown in blue while the stars are the results of the calculations. In the bottom row (**d–f**) red stars stand for the weights of the self-loops in the embedded network and black stars for the other edges. Note how the self-loops gain more weight as compared to the rest of edges as *q* is greater, reflecting the more localized structure of the embedding. In the case *q* = 1 (**c**,**d**), the expression for the realized populations and for the weights of the embedded network are simple functions of attractiveness and therefore we show them as curves.

Intuitively, *α* is the number of nodes that have been embedded through the random walk process. For a more detailed derivation, see the SI. See Fig. 3 (top row) for a comparison of the analytical solution and simulations for different values of *q*.

### Embedded network

We now turn to analytical description of the embedded network *G*_Γ_. We investigate average quantities of interest for the adjacency matrix **Γ** and the dependence of these quantities on *G*_*A*_, *G*_*B*_, and attractiveness *f*_*i*_ in network B. In the course of the assignment process, pairs of nodes that are connected by an edge in network A can be assigned to network B either synchronously or asynchronously. When two adjacent nodes in *G*_*A*_ are assigned *synchronously*, their assignments (their respective target nodes in network B) will be correlated through the random walk process. To account for synchronous assignment, we calculate an expected adjacency matrix ***ψ***. The synchronous assignment happens always between a node in A (primary node, the center node in Fig. 2) that is selected for its assignment to a node in B and its not-yet located neighbors in A (secondary nodes, the big empty circles in Fig. 2). Therefore the edge connecting the two nodes from A will connect the two locations of B to which both nodes are assigned, and this locations will be related to each other by the random walk that the secondary nodes perform. The information about the relation between these two locations in B will be stored in the adjacency matrix ***ψ*** (*ψ*_*ij*_ and represents the number of connections in A between the nodes from A assigned to locations *i* and *j* in B only due to the process just described, the solid edges in Fig. 2). When two adjacent nodes in *G*_*A*_ are assigned *asynchronously*, their assignments to their respective target nodes will be uncorrelated. To account for asynchronous assignment, we calculate the number of *stubs* ${\phi }_{i}$ that a node *i* in B attains during the assignment process and we construct an expected adjacency matrix given a random stub pairing $(\frac{{\phi }_{i}({t}^{\ast }){\phi }_{j}({t}^{\ast })}{{\sum }_{l}\,{\phi }_{l}({t}^{\ast })}(1-\frac{1}{2}{\delta }_{ij}))$. These stubs correspond to the dotted and dashed-dotted lines in Fig. 2; as the former ones correspond to edges in network A that connect nodes that have been assigned a location at a previous step and the latter ones correspond to unlocated second neighbors of the primary node selected for location assignment. See the S.I. for the complete derivation of the equations. In the end the adjacency matrix of the embedded network is described by the sum of both contributions as

6 $$\langle {{\rm{\Gamma }}}_{ij}\rangle ={\psi }_{ij}({t}^{\ast })+\frac{{\phi }_{i}({t}^{\ast }){\phi }_{j}({t}^{\ast })}{\sum _{l}\,{\phi }_{l}({t}^{\ast })}(1-\frac{1}{2}{\delta }_{ij}).$$

For the case $q\ne 0$, for which the full derivation is given in the SI, the resulting adjacency matrix of the embedded network is given by Eq. 6 with

7 $${\psi }_{ij}({t}^{\ast })=q\alpha {f}_{i}{\delta }_{ij}+q\alpha \{{\omega }_{ij}+{\omega }_{ji}\}(1-\frac{1}{2}{\delta }_{ij}),$$

8 $${\phi }_{i}({t}^{\ast })=(q\beta +\gamma ){f}_{i}+q\beta \sum _{l}\,{f}_{l}{[{\boldsymbol{\Omega }}(q)]}_{il},$$

where

$$\begin{array}{rcl}{\omega }_{ij} & = & {f}_{j}{[{\boldsymbol{\Omega }}(q)]}_{ij}\\ {[{\boldsymbol{\Omega }}(q)]}_{ij} & = & \mathop{\sum }\limits_{r\mathrm{=1}}^{\infty }\,{\mathrm{(1}-q)}^{r}{[{{\bf{C}}}^{r}]}_{ij}\\ \alpha  & = & {\int }_{0}^{{t}^{\ast }}\,{\langle k\rangle }_{A}^{\oplus }(t)dt\\ \beta  & = & {\int }_{0}^{{t}^{\ast }}\,[{\langle {k}^{2}\rangle }_{A}^{\oplus }(t)-{\langle k\rangle }_{A}^{\oplus }(t)+{\langle k\rangle }_{A}^{\oplus }(t){\langle k\rangle }_{A}^{\dagger }(t)]dt\\ \gamma  & = & {\int }_{0}^{{t}^{\ast }}\,{\langle k\rangle }_{A}^{\dagger }(t)dt=N{\langle k\rangle }_{A}-2\alpha -\beta \mathrm{.}\end{array}$$

For the case *q* = 1, the matrix **Ω**(*q*) vanishes. This matrix is the only element involving the topological information of network *B*. Thus in this case the embedded connections between different nodes in *B* are random as in the case where *q* = 0, but the difference is that now there are many more connections inside each target node. So for *q* = 1, putting everything together, Eq. 6 can be written as

9 $$\langle {{\rm{\Gamma }}}_{ij}\rangle ={f}_{i}\alpha {\delta }_{ij}+{f}_{i}{f}_{j}({N}_{A}{\langle k\rangle }_{A}-2\alpha )(1-\frac{1}{2}{\delta }_{ij}),$$

therefore depending only on the attractiveness of network *B* nodes and the average degree of network *A*.

For the case *q* = 0, the embedded network is given by Eq. 6 with

10 $${\psi }_{ij}({t}^{\ast })=\alpha ({f}_{i}{v}_{j}^{0}+{f}_{j}{v}_{i}^{0})(1-\frac{1}{2}{\delta }_{ij}),$$

11 $${\phi }_{i}({t}^{\ast })=\gamma {f}_{i}+\beta {v}_{i}^{0},$$

where ${v}_{i}^{0}$ is the *i*’th component of the leading eigenvector of the matrix ***C*** normalized using the *L*_1_-norm. See the SI for more details.

## Discussion

We have introduced a simple, tractable model of network embedding that regulates the extent of localization of the mapping with a single parameter *q*. The model can also be interpreted within the multilayer network framework, in which case networks A and B correspond to different layers and the assignment of source nodes in network A to target nodes in network B to specifying the interlayer connectivity structure^4^. We leave it as a future challenge to investigate likely biases in the model that are now induced by the fact that, at each step, two different types of location assignments are done: a source node from network A is assigned randomly to a target node in network B, while the unassigned neighbors of the source node are assigned to the neighborhood of the target location in B via a weighted random walk. For example, choosing a node from network A for location assignment in proportion to its degree may have nontrivial effects on the outcome of the assignment. We would like to stress that the simplicity of the model is what allows us to calculate some of its properties analytically, and as such it can help lay a foundation for the understanding dynamics that take place on embedded networks. We postulate our model thus as a tool for the theoretical exploration for example of epidemic dynamics on social networks that are embedded in geography and so gain a deeper understanding of spatial dynamics derived from purely network dynamics. Consider a situation where we have accurate representations of a social network and a spatial network, where the former is embedded in the latter, but no details are available on how the networks are inter-connected. In this case our model could be used as a null model for the embedding process based on values of the coupling parameter from settings where it is available or can be estimated from data. As a sensitivity analysis, one could then vary the value of this parameter to explore different degrees of locality. Finally as an intralayer connectivity model in the multilayer paradigm this model is also a useful tool in exploring the impact of the locality of the embedding on different settings.

## Supplementary information

Supplementary information for: Flexible model of network embedding
