# Supplementary material for: Flexible model of network embedding
Source: Sci Rep. 2019 Aug 12;9:11710. doi: 10.1038/s41598-019-48217-x (PMC6691014; doi:10.1038/s41598-019-48217-x)
Supplement: Supplementary file 1 — Supplementary information for: Flexible model of network embedding [file 41598_2019_48217_MOESM1_ESM.pdf]

Supplementary information for: Flexible model of network embedding

Juan Fernández-Gracia[1,2,*] **and Jukka-Pekka Onnela**[3]

[1]Department of Epidemiology, Harvard T.H. Chan School of Public Health, Harvard University, Boston, MA 02115, USA
[2]Instituto de Física Interdisciplinar y Sistemas Complejos IFISC (CSIC - UIB), Palma de Mallorca, E-07122, Spain
[3]Department of Biostatistics, Harvard T.H. Chan School of Public Health, Harvard University, Boston, MA 02115, USA
[*]juanf@ifisc.uib-csic.es

ABSTRACT

In this supplementary information we show the calculations that conclude in the results shown in the main text.

1 Step by step derivation of the model equations

1.1 Location assignment process

If the stopping probability q equals zero, we assign *location* in G_B to one node from G_A. If the parameter q differs from zero, we assign *location* in G_B to a random node and all of its neighbors with unassigned location in G_A. The description of the dynamics of this process will help us later for computing several quantities of the embedded network G_Γ. For a node in G_A to be assigned a *location* in G_B is independent of the assigned locations. At each (time) step of the process, we choose a random node from G_A that has not yet been assigned a location and will assign a location in G_B to the node together with all of its first (nearest) neighbors that do not yet have a location assigned. There are two distributions of interest: the distribution $P_A^{\oplus}(k,t)$ of the number of neighbors k of a node in G_A with unassigned location (empty circles in Fig. S1) for the nodes in G_A with unassigned location at a certain time step t, and the distribution $P_A^{\dagger}(k,t)$ of the number of neighbors k with assigned location (black small circles in Fig. S1) for the nodes in G_A with unassigned location at a certain time step t.

We first describe the dynamics of $P_A^{\oplus}(k,t)$, *i.e.*, the probability that an unlocated node has k unlocated neighbors at time t. We compute the dynamics of the number $\eta_k^{\oplus}(t)$ of unassigned nodes with k unlocated neighbors at time t and from that derive the equations for $P_A^{\oplus}(k,t) = \eta_k^{\oplus}(t)/\eta(t)$, where $\eta(t) = \sum_k \eta_k^{\oplus}(t)$ is the number of unlocated nodes at time t. The evolution of $\eta_k^{\oplus}(t)$ depends on three processes

1. The node chosen at random for location assignment, from now on called the *central node*, has k unlocated neighbors which happens with probability $P_A^{\oplus}(k,t)$ and reduces the number of nodes in the k category $\eta_k^{\oplus}$ by 1.

2. Some neighbors of the central node have k unlocated neighbors. If the central node has k_i unlocated neighbors, the probability of randomly choosing it is $P_A^{\oplus}(k_i,t)$, and the number of unlocated nodes with k unlocated neighbors that are neighbors of the central node is $k_i P_A^{\oplus}(k|k_i)$ (number by which $\eta_k^{\oplus}$ is reduced). $P_A(k|k_i)$ is the probability that an unlocated node with k unlocated neighbors is attached to the central node given that it has k_i unlocated neighbors. Finally we have to sum over all possible values of k_i.

3. A transition process. The second neighbors of the central node loose at least one link to an unlocated neighbor. Here we assume that the network is locally tree-like, so each second neighbor of the central node looses exactly one link. So this process will contribute in two ways: there is a loss for $\eta_k^{\oplus}$ if the second neighbor has k unlocated neighbors, while there is a gain if it has $k+1$ unlocated neighbors. We choose a central node of degree k_i with probability $P_A(k_i,t)$, the number of neighbors of degree k_j is $k_i P_A^{\oplus}(k_j|k_i)$, and finally the number of secondary neighbors of degree k $(k+1)$ is $k_j P_A^{\oplus}(k|k_j)$ $(k_j P_A^{\oplus}(k+1|k_j))$ represent a loss (gain) for $\eta_k^{\oplus}$. We have to sum over all possibilities of k_i and k_j.

The conditional probabilities also depend on time, but for clarity we have suppressed the temporal dependence in our notation. Putting everything together we have

$$\eta_k^{\oplus}(t+1) = \eta_k^{\oplus}(t) - P_A^{\oplus}(k,t) - \sum_{k_i} P_A^{\oplus}(k_i,t) k_i P_A^{\oplus}(k|k_i) - \sum_{k_i} P_A^{\oplus}(k_i,t) k_i \sum_{k_j} P_A^{\oplus}(k_j|k_i) k_j \left[P_A^{\oplus}(k|k_j) - P_A^{\oplus}(k+1|k_j) \right]. \quad (1)$$

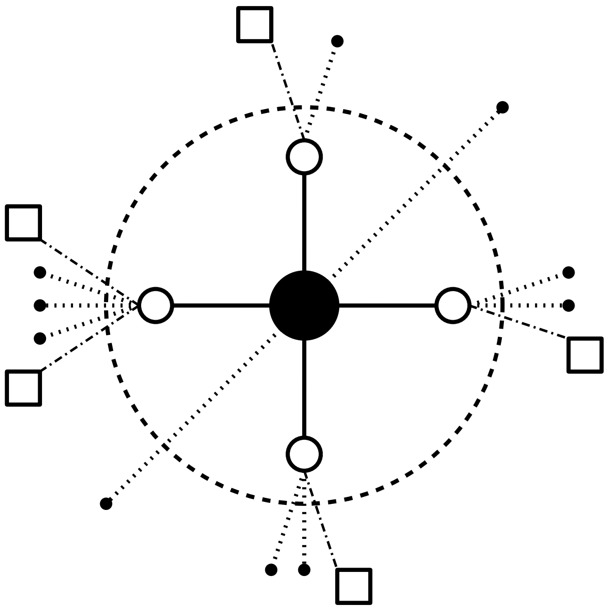

Figure S1. Schematic of the process of location assignment. Shown is a portion of the social network around the node which is chosen for location assignment at this time step. Only nodes up to the second neighborhood are shown. The black filled node in the middle represents the randomly chosen node for location assignment at a certain intermediate time step of the dynamics of location assignment. The empty circles represent the first neighbors of the central node which do not have an assigned location. The nodes inside the large dashed circle are the ones that will be assigned a location in this time step. The empty squares represent second neighbors of the central node with unassigned locations. Finally the small filled circles represent nodes that already have locations assigned.

By summing for all k we can see that the total number of unlocated nodes $\eta(t) = \sum_k \eta_k^{\oplus}(t)$ follows

$$\eta(t+1) = \eta(t) - [1 + \langle k \rangle_A^{\oplus}(t)], \tag{2}$$

with $\langle k \rangle_A^{\oplus}(t) = \sum_k k P_A^{\oplus}(k,t)$. For this result we have used the facts that $\sum_k P_A^{\oplus}(k,t) = 1$ and $\sum_k P_A^{\oplus}(k|k') = 1$, which are basic normalization properties.

We now return to the formulation of time-dependent probabilities by noting that $P_A^{\oplus}(k,t) = \eta_k^{\oplus}(t)/\eta(t)$, *i.e.*, its dynamical equation (map) is given by dividing Eq.(1) by Eq.(2). We now assume that the network is uncorrelated, which translates to the conditional probabilities

$$P_A^{\oplus}(k|k') = \frac{k}{\langle k \rangle_A^{\oplus}(t)} P_A^{\oplus}(k,t), \tag{3}$$

and therefore

$$P_A^{\oplus}(k,t+1) = \frac{1}{\eta(t) - 1 - \langle k \rangle_A^{\oplus}(t)} \left\{ P_A^{\oplus}(k,t)\,[\eta(t) - 1 - k] + \frac{\langle k^2 \rangle_A^{\oplus}(t)}{\langle k \rangle_A^{\oplus}(t)} \left[(k+1) P_A^{\oplus}(k+1,t) - k P_A^{\oplus}(k,t) \right] \right\}. \tag{4}$$

Now let us take the limit to continuous time. We consider $P_A^{\oplus}(k,t+1) - P_A^{\oplus}(k,t) \simeq \partial P_A^{\oplus}(k,t)/\partial t$ and end up with

$$\frac{\partial}{\partial t} P_A^{\oplus}(k,t) = \frac{1}{\eta(t) - 1 - \langle k \rangle_A^{\oplus}(t)} \left\{ P_A^{\oplus}(k,t) \left[\langle k \rangle_A^{\oplus}(t) - k \right] + \frac{\langle k^2 \rangle_A^{\oplus}(t)}{\langle k \rangle_A^{\oplus}(t)} \left[(k+1) P_A^{\oplus}(k+1,t) - k P_A^{\oplus}(k,t) \right] \right\}. \tag{5}$$

Note that this equation depends on the first two moments of the distribution $P_A^{\oplus}(k,t)$ and the number of nodes with unassigned locations $\eta(t)$. Also note that the initial condition is actually $P_A^{\oplus}(k,t=0) = P_A(k)$ as at time $t=0$ all nodes are unlocated. The moment of order m is defined as

$$\langle k^m \rangle_A^{\oplus}(t) = \sum_{k=0}^{\infty} k^m P_A^{\oplus}(k,t), \tag{6}$$

and we can obtain its dynamical equations by multiplying Eq.(5) by k^m and summing over k from 0 to ∞. Here we apply that sum over k. For the term on $(k+1)P_A^{\oplus}(k+1,t)$ we use the fact that

$$\sum_{k=0}^{\infty} k^m (k+1) P_A^{\oplus}(k+1,t) = \sum_{i=1}^{\infty} (i-1)^m i P_A^{\oplus}(i,t) \tag{7}$$

$$= \sum_{i=0}^{\infty} (i-1)^m i P_A^{\oplus}(i,t) \tag{8}$$

$$= \langle k(k-1)^m \rangle_A^{\oplus}(t). \tag{9}$$

Finally we end up with

$$\frac{d}{dt}\langle k^m \rangle_A^{\oplus}(t) = \frac{1}{\eta(t) - 1 - \langle k \rangle_A^{\oplus}(t)} \left\{ \langle k^m \rangle_A^{\oplus}(t) \langle k \rangle_A^{\oplus}(t) - \langle k^{m+1} \rangle_A^{\oplus}(t) + \frac{\langle k^2 \rangle_A^{\oplus}(t)}{\langle k \rangle_A^{\oplus}(t)} \left[\langle k(k-1)^m \rangle_A^{\oplus}(t) - \langle k^{m+1} \rangle_A^{\oplus}(t) \right] \right\}. \tag{10}$$

Note that this forms an infinite hierarchy of equations, as the equation for the moment of order m depends on itself, all lower order moments and the moment of order $m+1$. In this type of situation a typical approach is to use a moment closure procedure, which approximates higher order moments by lower order ones. Here we have moments 1 and 2 independent and close the hierarchy by approximating the moment of order 3. One possible approach is to set higher order cumulants to zero. Truncating at order two, the third moment is then given by

$$\langle k^3 \rangle_S^{\oplus}(t) = 3\langle k \rangle_S^{\oplus}(t) \langle k^2 \rangle_S^{\oplus}(t) - 2\langle k \rangle_S^{\oplus}(t)^3. \tag{11}$$

By truncating at second order we set the third cumulant $\kappa_3 = 0$ and thus imply that the distribution is symmetric as the skewness of a distribution is proportional to its third cumulant. A way around this is to approximate the third moment by its function of the first two if the distribution were lognormal, which yields

$$\langle k^3 \rangle_S^{\oplus}(t) = \left(\frac{\langle k^2 \rangle_S^{\oplus}(t)}{\langle k \rangle_S^{\oplus}(t)^2} + 2 \right) \langle k \rangle_S^{\oplus}(t)^3 \left(\frac{\langle k^2 \rangle_S^{\oplus}(t)}{\langle k \rangle_S^{\oplus}(t)^2} - 1 \right)^2 + 2\langle k \rangle_S^{\oplus}(t) \langle k^2 \rangle_S^{\oplus}(t) - \langle k \rangle_S^{\oplus}(t)^3 \tag{12}$$

A numerical investigation showed that both prescriptions for moment closure give very similar results. Besides that, by passing to the continuum in Eq.(2), we have a closed system of three coupled ordinary differential equations, namely for the first two moments of $P_S^{\oplus}(k,t)$ and the number of remaining nodes without location $\eta(t)$.

$$\frac{d}{dt}\eta(t) = -\left[1 + \langle k \rangle_A^{\oplus}(t)\right], \tag{13}$$

$$\frac{d}{dt}\langle k \rangle_A^{\oplus}(t) = \frac{1}{\eta(t) - 1 - \langle k \rangle_A^{\oplus}(t)} \left[\langle k \rangle_A^{\oplus}(t)^2 - 2\langle k^2 \rangle_A^{\oplus}(t) \right], \tag{14}$$

$$\frac{d}{dt}\langle k^2 \rangle_A^{\oplus}(t) = \frac{1}{\eta(t) - 1 - \langle k \rangle_A(t)} \left\{ \langle k^2 \rangle_A^{\oplus}(t) \langle k \rangle_A^{\oplus}(t) + \frac{\langle k^2 \rangle_A^{\oplus}(t)}{\langle k \rangle_A^{\oplus}(t)} \left[\langle k \rangle_A^{\oplus}(t) - 2\langle k^2 \rangle_A^{\oplus}(t) \right] - \langle k^3 \rangle_A^{\oplus}(t) \right\}. \tag{15}$$

The initial conditions are given by

$$\eta(t=0) = N_A \quad , \quad \langle k^m \rangle_A^{\oplus}(t=0) = \sum_k k^m P_A(k). \tag{16}$$

Note also that after t^* we will have assigned location to all nodes, which means $\eta(t^*) = 0$. Then by integrating the equation for $\eta(t)$ between $t = 0$ and $t = t^*$

$$N_A = \int_0^{t^*} \left[1 + \langle k \rangle_A^{\oplus}(t)\right] dt. \tag{17}$$

For the distribution $P_A^{\dagger}(k,t)$ of unlocated nodes in G_A with k located nodes at time step t, we proceed similarly to the previous case, describing the dynamics of the number $\eta_k^{\dagger}(t)$ (rather than than the proportion) and then approximate the distribution by the proportions. Note that $\sum_k \eta_k^{\dagger}(t) = \sum_k \eta_k^{\oplus}(t) = \eta(t)$ as both sums equal the number of unlocated nodes. There are again three processes by which the number $\eta_k^{\dagger}(t)$ changes:

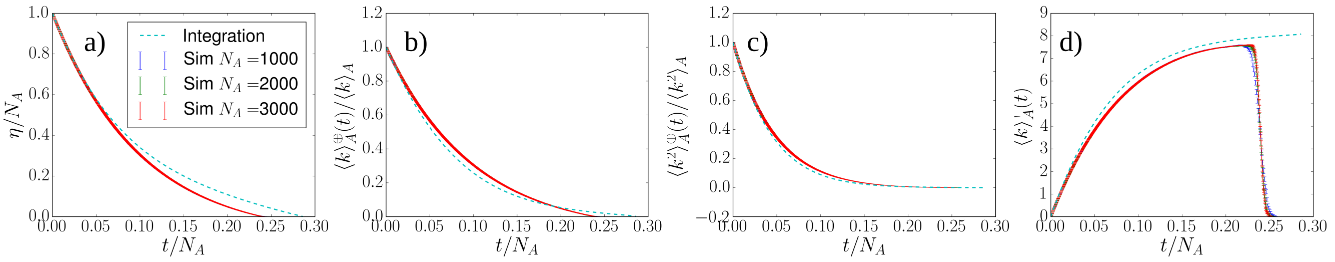

Figure S2. (Color online) **Assignment results.** We simulated 1000 independent realizations of Erdös-Renyi networks of different sizes ($N_A = 1000$ in blue, 2000 in green and 3000 in red). Cyan dashed lines for the results of integrating Eqs. 13– 15 and Eq. 21. The x-axis shows the time t normalized by the size of the network, N_A. **a)** Proportion of unlocated nodes $\eta(t)/N_A$. **b)** Average number of unlocated neighbors for unlocated nodes divided by the average degree of nodes in network A, $\langle k\rangle_A^{\oplus}/\langle k\rangle_A$. **c)** Second moment of the number of unlocated neighbors for unlocated nodes divided by the second moment of the degree distribution of network A, $\langle k^2\rangle_A^{\oplus}/\langle k^2\rangle_A$. **d)** Average number of located neighbors for unlocated nodes $\langle k\rangle_A^{\dagger}$.

1. $\eta_k^{\dagger}(t)$ decreases by one if the central node chosen for location assignment has k located neighbors. We choose a central node with k located neighbors with probability $P_A^{\dagger}(k,t)$.
2. $\eta_k^{\dagger}(t)$ decreases by the number of unlocated neighbors of the central node that have k located neighbors. With probability $P_A^{\oplus}(k_i,t)$ the central node has k_i unlocated neighbors and all of them have a probability $P_A^{\dagger}(k,t)$ of having k located neighbors. Finally this term will be summed over all possible values of k_i.
3. There is a transition process. The unlocated second neighbors (second order neighbors via unlocated first order neighbors) of the central node gain one located neighbor, therefore the number of those which have k unlocated neighbors will decrease $\eta_k^{\dagger}(t)$, while the ones with $k-1$ unlocated neighbors will increase it. Here we again assume that the network is locally tree-like.

Putting everything together we end up with

$$\eta_k^{\dagger}(t+1) = \eta_k^{\dagger}(t) - P_A^{\dagger}(k,t) - \sum_{k_i} P_A^{\oplus}(k_i,t) k_i P_A^{\dagger}(k,t) + \sum_{k_i} P_A^{\oplus}(k_i,t) k_i \sum_{k_j} P_A^{\oplus}(k_j|k_i) k_j \left[P_A^{\dagger}(k-1,t) - P_A^{\dagger}(k,t) \right]. \tag{18}$$

Assuming that the network is uncorrelated, using that $P_A^{\dagger}(k,t) = \eta_k^{\dagger}(t)/\eta(t)$ and passing to the time continuum limit, we get

$$\frac{\partial}{\partial t} P_A^{\dagger}(k,t) = \frac{\langle k^2\rangle_A^{\oplus}(t)}{\eta(t) - 1 - \langle k\rangle_A^{\oplus}(t)} \left[P_A^{\dagger}(k-1,t) - P_A^{\dagger}(k,t) \right]. \tag{19}$$

Here the initial condition is given by $P_A^{\dagger}(k,t=0) = \delta_{k0}$ since at $t=0$ all nodes are unlocated and thus no node has any located neighbor. Again we can find the dynamical equation for the moment of order m, $\langle k^m\rangle_A^{\dagger}(t)$, by multiplying the previous equation by k^m and summing over all k which yields

$$\frac{d}{dt} \langle k^m\rangle_A^{\dagger}(t) = \frac{\langle k^2\rangle_A^{\oplus}(t)}{\eta(t) - 1 - \langle k\rangle_A^{\oplus}(t)} \left[\langle (k+1)^m\rangle_A^{\dagger}(t) - \langle k^m\rangle_A^{\dagger}(t) \right]. \tag{20}$$

Note that the equation for the moments forms a closed system of any maximum order as the equation for the moment of order m does not depend on higher order moments. The initial condition translates for the moments in $\langle k^m\rangle_A^{\dagger}(t=0) = 0$. In fact, for the calculations later in the text we only need the first moment whose dynamics are described by

$$\frac{d}{dt} \langle k\rangle_A^{\dagger}(t) = \frac{\langle k^2\rangle_A^{\oplus}(t)}{\eta(t) - 1 - \langle k\rangle_A^{\oplus}(t)}. \tag{21}$$

1.2 Realized sizes of node populations

We use $\Phi_i(t)$ to denote the number of nodes in the social network that have been assigned location i. In the $q \neq 0$ case, the average realized populations $\langle \Phi_i\rangle(t)$ grow per time step due to two different mechanisms. The first mechanism is that the

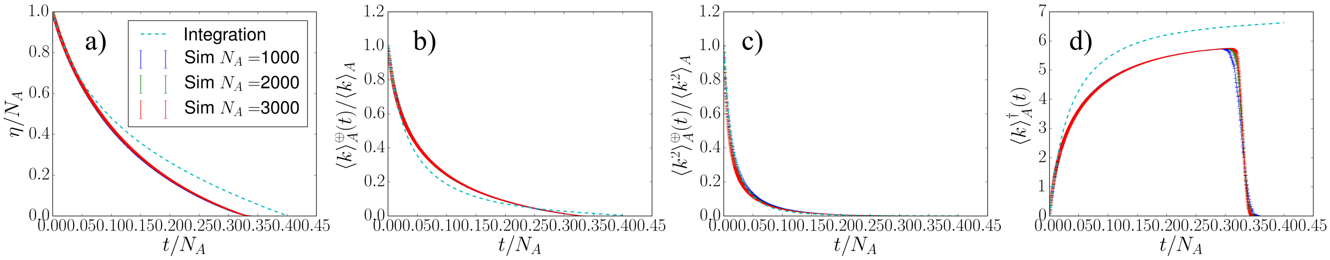

Figure S3. (Color online) **Location assignment process: agreement between simulations and calculations.** We simulated 1000 independent realizations of Barabási-Albert networks of different sizes ($N_A = 1000$ in blue, 2000 in green and 3000 in red). Cyan dashed lines for the results of integrating Eqs.A14–A15 and A21. The x-axis shows the time t normalized by the size of the network, N_A. **a)** Fraction of unlocated nodes $\eta(t)/N_A$. **b)** Average number of unlocated neighbors for unlocated nodes divided by the average degree of nodes in network A, $\langle k\rangle_A^{\oplus}/\langle k\rangle_A$. **c)** Second moment of the number of unlocated neighbors for unlocated nodes divided by the second moment of the degree distribution of network A, $\langle k^2\rangle_A^{\oplus}/\langle k^2\rangle_A$. **d)** Average number of located neighbors for unlocated nodes $\langle k\rangle_A^{\dagger}$.

central node in the location assignment process is assigned location node i, what happens with probability f_i. The second mechanism is due to unlocated first neighbors of the central node being assigned location i. This second mechanism in principle can have infinitely many terms, as many as there are steps in the random walk from G_A to G_B. The equation (map) for the average values $\langle\Phi_i\rangle(t)$ is

$$\begin{aligned}\langle\Phi_i\rangle(t+1) = &\langle\Phi_i\rangle(t) + f_i + f_i\sum_k P_A^{\oplus}(k,t)kq + \sum_j f_j \sum_k P_A^{\oplus}(k,t)k(1-q)\frac{f_i B_{ij}}{\sum_l f_l B_{lj}}q \\ &+\sum_j f_j \sum_k P_A^{\oplus}(k,t)k(1-q)\sum_m \frac{f_m B_{mj}}{\sum_l f_l B_{lj}}(1-q)\frac{f_i B_{im}}{\sum_l f_l B_{lm}}q \\ &+\sum_j f_j \sum_k P_A^{\oplus}(k,t)k(1-q)\sum_m \frac{f_m B_{mj}}{\sum_l f_l B_{lj}}\sum_n (1-q)\frac{f_n B_{nm}}{\sum_l f_l B_{lm}}(1-q)\frac{f_i B_{in}}{\sum_l f_l B_{ln}}q \\ &+\dots \end{aligned} \tag{22}$$

Here B_{ij} correspond the elements of the adjacency matrix of G_B. We define the matrix C with elements

$$C_{ij} = \frac{f_i}{\sum_l f_l B_{lj}} B_{ij}, \tag{23}$$

which specify the probability of a single jump of the random walk starting at location j and ending at location i, where the actual jump happens with probability $1-q$). It is straightforward to show that the matrix C has the normalization property $\sum_i C_{ij} = 1$, which is the probability of jumping from j to any other location linked in the geographical network. This matrix describes a weighted directed network with the same underlying undirected topology as the geographical network B.
Now using this matrix we can rewrite Eq. 22, and in the continuous time limit we have

$$\frac{d}{dt}\langle\Phi_i\rangle(t) = f_i\left[1 + q\langle k\rangle_A^{\oplus}(t)\right] + q\langle k\rangle_A^{\oplus}(t)\sum_j f_j \sum_{r=1}^{\infty}(1-q)^r \left[C^r\right]_{ij}, \tag{24}$$

where $\left[C^r\right]_{ij}$ is the element ij of the rth power of matrix C. This element gives the probability of a random walk of distance r from j to i given that the random walk consists of r steps.
The initial condition for Eq. 24 is $\langle\Phi_i\rangle(t=0) = 0$ because there are no located nodes at $t = 0$. After t^* time steps all nodes have been located and the average realized population sizes follow

$$\langle\Phi_i\rangle(t^*) = f_i\int_0^{t^*}\left[1 + q\langle k\rangle_A^{\oplus}(t)\right]dt + q\left(\int_0^{t^*}\langle k\rangle_A^{\oplus}(t)dt\right)\sum_j f_j \sum_{r=1}^{\infty}(1-q)^r\left[C^r\right]_{ij}, \quad (q \neq 0) \tag{25}$$

which is obtained by integrating Eq. 24.
In the case $q = 1$, combining Eqs. 25 and 17, we see that $\langle\Phi_i\rangle(t^*) = N_A f_i$ and, as in the $q = 0$ case, the realized population

sizes are have the same proportions as the input populations.
For intermediate values of the stopping probability q, the random walk on the network distorts the realized populations as described by Eq. 25.

For the case $q = 0$ we are going to assign first one random source node α of network A to a target node i in network B proportionally to the attractiveness f_i of node i. The unassigned neighbors of α will be assigned to a node j in network B proportionally the stationary probability of the weighted random walk. This will be proportional to the corresponding entry of the leading eigenvector v_j^0 of the matrix C, which is the one encoding the transition probabilities in the random walk. One can also see this from Eq. 22. In that equation from the third term on on the right hand side, each term describes the random walk of different lengths, stopping at a certain point. Because it is stopping, each term has a factor of q, and therefore for $q = 0$ they disappear. This is true except for the term at infinity. Therefore the equation is substituted by

$$\frac{d}{dt}\langle\Phi_i\rangle(t) = f_i + \langle k\rangle_A^{\oplus}(t)\sum_j f_j \lim_{r\to\infty}[C^r]_{ij}. \tag{26}$$

Now we note that the powers of C converge such that $\lim_{r\to\infty}[C^r]_{ij} = v_i^0$, and as the attractivenesses are normalized, after integrating, we get

$$\langle\Phi_i\rangle(t^*) = f_i t^* + \alpha v_i^0 \tag{27}$$

$$= f_i(N_A - \alpha) + \alpha v_i^0, \quad (q \neq 0) \tag{28}$$

with $\alpha = \int_0^{t^*}\langle k\rangle_A^{\oplus}(t)dt$. Here we are using also that the eigenvector is normalized so that $\sum_i v_i^0 = 1$, *i.e.*, wirh an L_1-norm. Similar reasoning as the one presented above will be used to derive further quantities in the case $q = 0$.

1.3 Embedded network

Now we turn to the analytical description of the embedded network G_Γ. Again we describe the form of the average for those quantities for the adjacency matrix Γ, and their dependence on the inputs G_A, G_B and attractiveness f_i).
At each time step, the processes by which two nodes in G_Γ (this set of nodes is exactly equal to the set of nodes of G_B) i and j (which could be the same) gain connections are the following:

1. The links that connect the central node to unlocated neighbors. We can describe this phenomenon through the process of the random walk of the first neighbor and how this process contributes to the number of connections between nodes i and j. We use $\psi_{ij}(t)$ to denote the number of connections due to this process at time t. These are the solid links in Fig. S1.

2. Node i gains links to an uncorrelated location. For this process, we count how many *stubs* are accumulated by each node and we approximate how they are linked to other nodes under a random pairing of stubs. We use $\varphi_i(t)$ to denote the total number of these stubs connected to node i at time t. This process can happen in two ways ($\varphi_i(t) = \zeta_i(t) + \xi_i(t)$).

   (a) Let $\zeta_i(t)$ denote the number of stubs gained by node i up to time t due to neighbors of the central node being located to i. These nodes will have links to nodes that are at present unlocated. These are the dashed-dotted links in Fig. S1.

   (b) Let $\xi_i(t)$ denote the number of stubs gained by location i up to time t due to having nodes located there that have links to already located nodes. These are the dotted links in Fig. S1.

Finally, the average number of social connections will be

$$\langle\Gamma_{ij}\rangle = \psi_{ij}(t^*) + \frac{\varphi_i(t^*)\varphi_j(t^*)}{\sum_l \varphi_l(t^*)}\left(1 - \frac{1}{2}\delta_{ij}\right) \tag{29}$$

In the $q \neq 0$ case, the number of correlated connections (solid edges in Fig. S1) between nodes i and j will be equal to the probability of locating the central node at i multiplied by the probability of an unlocated neighbor of the central node stopping its random walk at node j. We then need to sum the same probability inverting i and j, which results in

$$\psi_{ij}(t+1) = \psi_{ij}(t) + q\sum_k P_A^{\oplus}(k,t)k\left\{f_i\delta_{ij} + \left[f_i\sum_{r=1}^{\infty}(1-q)^r[C^r]_{ji} + f_j\sum_{r=1}^{\infty}(1-q)^r[C^r]_{ij}\right]\left(1-\frac{1}{2}\delta_{ij}\right)\right\} \tag{30}$$

$$= \psi_{ij}(t) + q\langle k\rangle_A^{\oplus}(t)\left\{f_i\delta_{ij} + \left[\sum_{r=1}^{\infty}(1-q)^r\left(f_i[C^r]_{ji} + f_j[C^r]_{ij}\right)\right]\left(1-\frac{1}{2}\delta_{ij}\right)\right\}. \tag{31}$$

The term $(1-\frac{1}{2}\delta_{ij})$ corrects for the fact that without it we would be double-counting when $i=j$. Passing to the time continuum we obtain

$$\frac{d}{dt}\psi_{ij}(t) = q\langle k\rangle_A^{\oplus}(t)\left\{f_i\delta_{ij} + \left[\sum_{r=1}^{\infty}(1-q)^r\left(f_i\,[C^r]_{ji} + f_j\,[C^r]_{ij}\right)\right]\left(1-\frac{1}{2}\delta_{ij}\right)\right\}. \tag{32}$$

Now integrating from $t=0$ to $t=t^*$, with the condition $\psi_{ij}(t=0)=0$, we obtain

$$\psi_{ij}(t^*) = q\int_0^{t^*}\langle k\rangle_A^{\oplus}(t)dt\left\{f_i\delta_{ij} + \left[\sum_{r=1}^{\infty}(1-q)^r\left(f_i\,[C^r]_{ji} + f_j\,[C^r]_{ij}\right)\right]\left(1-\frac{1}{2}\delta_{ij}\right)\right\}. \tag{33}$$

Now for $\zeta_i(t)$, the number of stubs gained by node i up to time t due to neighbors of the central node being located to node i with unlocated neighbors, we have

$$\zeta_i(t+1) = \zeta_i(t) + q\left\{f_i\sum_k P_A^{\oplus}(k,t)k\sum_{k'}P_A^{\oplus}(k'|k)(k'-1) + \sum_j f_j\sum_k P_A^{\oplus}(k,t)k\sum_{k'}P_A^{\oplus}(k'|k)(k'-1)\sum_{r=1}^{\infty}(1-q)^r\,[C^r]_{ij}\right\} \tag{34}$$

$$= \zeta_i(t) + q\left[\langle k^2\rangle_A^{\oplus}(t) - \langle k\rangle_A^{\oplus}(t)\right]\left\{f_i + \sum_j f_j\sum_{r=1}^{\infty}(1-q)^r\,[C^r]_{ij}\right\}. \tag{35}$$

Again, passing to a continuum we have

$$\frac{d}{dt}\zeta_i(t) = q\left[\langle k^2\rangle_A^{\oplus}(t) - \langle k\rangle_A^{\oplus}(t)\right]\left\{f_i + \sum_j f_j\sum_{r=1}^{\infty}(1-q)^r\,[C^r]_{ij}\right\}. \tag{36}$$

Now integrating and bearing in mind the initial condition $\zeta_i(t=0)=0$

$$\zeta_i(t^*) = q\int_0^{t^*}\left[\langle k^2\rangle_A^{\oplus}(t) - \langle k\rangle_A^{\oplus}(t)\right]dt\left\{f_i + \sum_j f_j\sum_{r=1}^{\infty}(1-q)^r\,[C^r]_{ij}\right\}. \tag{37}$$

Finally, for $\xi_i(t)$, the number of stubs gained by location i up to time t due to having nodes located there that have links to previously located nodes, we have

$$\xi_i(t+1) = \xi_i(t) + f_i\sum_k P_A^{\dagger}(k,t)k + qf_i\sum_k P_A^{\oplus}(k,t)k\sum_{k'}P_A^{\dagger}(k',t)k' + \dots \tag{38}$$

$$\dots + q\sum_j f_j\sum_k P_A^{\oplus}(k,t)k\sum_{k'}P_A^{\dagger}(k',t)k'\sum_{r=1}^{\infty}(1-q)^r\,[C^r]_{ij}$$

$$= \xi_i(t) + f_i\langle k\rangle_A^{\dagger}(t)\left[1+q\langle k\rangle_A^{\oplus}(t)\right] + q\langle k\rangle_A^{\oplus}(t)\langle k\rangle_A^{\dagger}(t)\sum_j f_j\sum_{r=1}^{\infty}(1-q)^r\,[C^r]_{ij}. \tag{39}$$

Treating time as continuous, and integrating from $t=0$ to $t=t^*$ with the initial condition $\xi_i(t=0)=0$, yields

$$\xi_i(t^*) = f_i\int_0^{t^*}\langle k\rangle_A^{\dagger}(t)dt + q\int_0^{t^*}\langle k\rangle_A^{\oplus}(t)\langle k\rangle_A^{\dagger}(t)dt\left\{f_i + \sum_j f_j\sum_{r=1}^{\infty}(1-q)^r\,[C^r]_{ij}\right\}. \tag{40}$$

So, summing up, in the general case we have the equation (29) with

$$\psi_{ij}(t^*) = q\alpha f_i\delta_{ij} + q\alpha\left\{f_i\,[\Omega(q)]_{ji} + f_j\,[\Omega(q)]_{ij}\right\}\left(1-\frac{1}{2}\delta_{ij}\right), \tag{41}$$

$$\varphi_i(t^*) = (q\beta+\gamma)f_i + q\beta\sum_l f_l\,[\Omega(q)]_{il}, \tag{42}$$

$$\sum_i\varphi_i = \beta+\gamma = N_A\langle k\rangle_A - 2\alpha, \tag{43}$$

where

$$[\Omega(q)]_{ij} = \sum_{r=1}^{\infty} (1-q)^r [C^r]_{ij}, \tag{44}$$

$$\alpha = \int_0^{t^*} \langle k \rangle_A^{\oplus}(t) dt, \tag{45}$$

$$\beta = \int_0^{t^*} \left[\langle k^2 \rangle_A^{\oplus}(t) - \langle k \rangle_A^{\oplus}(t) + \langle k \rangle_A^{\oplus}(t) \langle k \rangle_A^{\dagger}(t) \right] dt, \tag{46}$$

$$\gamma = \int_0^{t^*} \langle k \rangle_A^{\dagger}(t) dt = N \langle k \rangle_A - 2\alpha - \beta. \tag{47}$$

For Eq.(43) note that we count all stubs of edges whose ends were not assigned a location during that same time step.

For the $q = 1$ case, note that the last terms inside curly brackets in Eqs. 33, 37 and 40 vanish. Those terms are the only ones involving geographical network information. Thus, in this case, the embedded connections between different locations are random as in the $q = 0$ case. The difference now is that there are many more social connections inside the same location. So for $q = 1$, putting everything together, Eq. 29 is written as

$$\langle \Gamma_{ij} \rangle = f_i \alpha \delta_{ij} + f_i f_j (N_A \langle k \rangle_A - 2\alpha) \left(1 - \frac{1}{2} \delta_{ij} \right), \quad (q = 1). \tag{48}$$

Following similar reasoning as with the calculation of the realized populations for the case $q = 0$, one can derive that the embedding network in this case will be Eq. 29 with

$$\psi_{ij}(t^*) = \alpha \left(f_i v_j^0 + f_j v_i^0 \right) \left(1 - \frac{1}{2} \delta_{ij} \right), \tag{49}$$

$$\varphi_i(t^*) = \gamma f_i + \beta v_i^0, \tag{50}$$

where v_i^0 is the i'th component of leading eigenvector of the matrix C normalized with an L_1-norm.

2 Proportion of correlated embedded connections

We can compute the fraction ρ of all links of the social network whose end locations are correlated through the random walk:

$$\rho = \frac{\int_0^{t^*} \langle k \rangle_A^{\oplus}(t) dt}{N_A \langle k \rangle_A} = \frac{\alpha}{N_A \langle k \rangle_A}. \tag{51}$$

3 Possible mechanisms for enhancing the extent of correlations

The proportion of links with correlated locations can be increased by choosing the central node to be located in proportion to the number of unlocated neighbors. In this case we would have to recalculate the evolution equations and other quantities dealt with above. The proportion of correlated links would now be given by

$$\rho = \frac{\int_0^{t^*} \frac{\langle k^2 \rangle_A^{\oplus}(t)}{\langle k \rangle_A^{\oplus}(t)} dt}{N_A \langle k \rangle_A}. \tag{52}$$

Another possibility is to locate not only the first neighborhood of the central node, but also further neighborhoods. We anticipate that this would further complicate the analytical treatment of the quantities of interest.
